# The Treatment of Frost-Bite and of Burns

**Published:** 1918-02

**Authors:** 


***The Treatment of Frost-Bite and of Burns***. By F. RATHERY and L. BAUZIL. Trans. of Abs. in the *Archives de Médecine et de Pharmacie Militaires*, t. LXVII, N° 3, March 1917, p. 412, of an article in the *Paris Médical*, t. VII, N° 33, March 31, 1917, p. 249.

The primary object of this treatment is to reduce pain and also to permit of healing with the least possible delay and with a minimum loss of tissue.

The patient is first carefully washed with soap and boiling water, but in such a manner as not to remove the epidermis raised by the blisters. After drying, the lesion is swabbed by means of sterilized forceps with the following preparation, first melted in a double-boiler and kept at as high a temperature as the patient can bear :

| | |
|---|---|
| Naphtholate of soda . . . . . . . . . | 2 gm. |
| Essence of thyme . . . . . . . . . . . | 3 gm. each. |
| Essence of marjoram . . . . . . . . . | 3 gm. each. |
| Essence of geranium . . . . . . . . . | 3 gm. each. |
| Pure vasseline . . . . . . . . . . . | 1,000 gm. |
| Paraffin at 45°-50° C . . . . . . . . . | 5,000 gm. |

After fusion, distribute in 125 gm. vessels and sterilize for 20 minutes at 120° C.

The layer formed over the lesions by this preparation is in turn covered with a thin wad of sterilized cotton, and another application of the preparation is made on top of the cotton to make sure that the dressing is completely closed. More cotton is placed over the whole dressing and bandages applied.

At the start, the dressings should be renewed each day, taking care to irrigate the affected region with a warm sterilized artificial serum. The lesions should then be dried without rubbing, by means of a gauze compress.

As soon as the inflammation is lessened and healing begins, the dressings may be applied less frequently. When the epidermis appears, the treatment may be suspended, and the affected parts powdered with the following preparation :

| | |
|---|---|
| Camphor | 20 gm. |
| Borate of soda | 200 gm. |
| Talc | 200 gm. |

During the treatment it is unnecessary to pay any attention to the abundant and fetid discharges from the lesions, or to the often excessive granulation.

Although the authors have applied the cure in only a small number of cases, they report the following encouraging results :

1. The dressing is non-adherent and may easily be removed without injuring the epidermis.
2. All pain is stopped after the first application.
3. The edema and inflammation are almost immediately reduced.
4. Anatomical lesions are limited.
5. The wound quickly assumes a favorable appearance, and epidermis begins to form in small patches.
6. Healing is quickly effected without bad scars.

As a consequence of these favorable results the treatment has been extended to burns, and has given complete satisfaction.

